# Acute Zonal Occult Outer Retinopathy in Japanese Patients: Clinical Features, Visual Function, and Factors Affecting Visual Function

**DOI:** 10.1371/journal.pone.0125133

**Published:** 2015-04-28

**Authors:** Saho Saito, Wataru Saito, Michiyuki Saito, Yuki Hashimoto, Shohei Mori, Kousuke Noda, Kenichi Namba, Susumu Ishida

**Affiliations:** 1 Department of Ophthalmology, Hokkaido University Graduate School of Medicine, Sapporo, Hokkaido, Japan; 2 Department of Ocular Circulation and Metabolism, Hokkaido University Graduate School of Medicine, Sapporo, Hokkaido, Japan; Saitama Medical University, JAPAN

## Abstract

**Purpose:**

To evaluate the clinical features and investigate their relationship with visual function in Japanese patients with acute zonal occult outer retinopathy (AZOOR).

**Methods:**

Fifty-two eyes of 38 Japanese AZOOR patients (31 female and 7 male patients; mean age at first visit, 35.0 years; median follow-up duration, 31 months) were retrospectively collected: 31 untreated eyes with good visual acuity and 21 systemic corticosteroid-treated eyes with progressive visual acuity loss. Variables affecting the logMAR values of best-corrected visual acuity (BCVA) and the mean deviation (MD) on Humphrey perimetry at initial and final visits were examined using multiple stepwise linear regression analysis.

**Results:**

In untreated eyes, the mean MD at the final visit was significantly higher than that at the initial visit (*P* = 0.00002). In corticosteroid-treated eyes, the logMAR BCVA and MD at the final visit were significantly better than the initial values (*P* = 0.007 and *P* = 0.02, respectively). The final logMAR BCVA was 0.0 or less in 85% of patients. Variables affecting initial visual function were moderate anterior vitreous cells, myopia severity, and a-wave amplitudes on electroretinography; factors affecting final visual function were the initial MD values, female sex, moderate anterior vitreous cells, and retinal atrophy.

**Conclusions:**

Our data indicated that visual functions in enrolled patients significantly improved spontaneously or after systemic corticosteroids therapy, suggesting that Japanese patients with AZOOR have good visual outcomes during the follow-up period of this study. Furthermore, initial visual field defects, gender, anterior vitreous cells, and retinal atrophy affected final visual functions in these patients.

## Introduction

Acute zonal occult outer retinopathy (AZOOR), first described by Gass in 1993, is an idiopathic syndrome of acute outer retinal impairment [1]. AZOOR is characterized by funduscopically normal or minimally abnormal retinal appearances at the early stage [1,2], which may occasionally be followed by retinal pigment epithelium (RPE) degeneration in the affected area [2,3].

Visual field defects in AZOOR patients are caused by outer retinal impairment which is demonstrated by impaired responses in multifocal electroretinography (ERG) [4] and a disrupted ellipsoid zone (originally called the inner segment/outer segment junction) detected by optical coherence tomography (OCT) [5,6], corresponding to AZOOR retinal lesions. Associated RPE damage can be more clearly visible by fundus autofluorescence (FAF) [3,7].

Regarding the pathogenesis, we recently demonstrated using laser speckle flowgraphy that the choroidal blood flow velocity at AZOOR lesions significantly increased as visual function and outer retinal morphology improved [8]. Changes in the blood flow showed “inflammatory” pattern in the choroid, similar to our previous findings in patients with choroiditis, Vogt-Koyanagi-Harada disease [9], and serpiginous choroiditis [10]. From these observations, we hypothesized that inflammation in the choroid caused secondary photoreceptor impairment in AZOOR and may explain the absent or minimally abnormal retinal appearance during early stages of AZOOR, although an alternative explanation is considered as follows; the outer retina is primarily affected by inflammation and destruction of photoreceptor outer segments leads to secondary reduction of the choroidal blood flow [11].

In 2002, Gass et al. reported the following clinical features of 51 primarily Caucasian AZOOR patients over more than three years of follow-up: young myopic women predominated; 76% had bilateral involvement at the final visit; the recurrence rate was 31%; subsequent retinal atrophy was observed in 48% of patients; visual field impairment ceased in 78% of patients 6 months after the initial visit, but partially improved in only 24% of patients; and the logarithm of minimal angle of resolution (logMAR) value of the best-corrected visual acuity (BCVA) at the final visit was 1.0 or more in 27% of patients [2]. These results showed that AZOOR patients did not have a good visual prognosis. Few studies examining the clinical characteristics or prognostic factors in a large case series have since been reported because of the condition’s rarity.

Only several single case reports and a few case series with small patient populations have described visual outcomes in Asian patients with AZOOR [12]. Among 13 Chinese AZOOR patients, the final logMAR BCVA was less than 0.35 in 57% of patients and more than 1.0 in only one patient [13]. However, in a study examining six Japanese patients, the final logMAR BCVA was 1.0 or more in 50% of the patients [14]. Apparently, AZOOR progression is variable [12], and reports of patients with good visual outcomes improving spontaneously [8,13,15,16] or showing poor visual prognosis [14,17] have been described. Thus, the visual prognosis in Asian patients with AZOOR remains to be elucidated. The present study evaluated the clinical features of AZOOR in Japanese patients, with the goal of determining factors affecting visual functions.

## Materials and Methods

### Subjects

In total, 52 eyes of 38 patients diagnosed with AZOOR who visited the vitreo-retina clinic at Hokkaido University Hospital from 2002 to 2012 and were followed up for more than six months were enrolled and their records were retrospectively reviewed. The study was approved by the ethics committee of Hokkaido University Hospital (approval ID: 014–0042) and followed the tenets of the Declaration of Helsinki. Written informed consent was obtained from all subjects after the nature and possible consequences of the study had been explained. AZOOR was diagnosed using the following criteria [2]: acute visual field or vision loss usually with concurrent photopsia; one or more visual field defect regions that could not be explained by funduscopic examination or fluorescein angiography (FA); decreased multifocal ERG responses corresponding to the retinal sites with visual field defects; and, beginning in 2007, outer retinal morphologic abnormalities including an absent or discontinuous ellipsoid zone on OCT [5,6,18]. Patients who involved funduscopic and angiographic findings consistent with white dots observed in AZOOR complex (e.g., multiple evanescent white dot syndrome) were excluded. Patients aged over 50 years or with medical history of uncontrolled systemic hypertension at disease onset were also excluded so that cancer-associated retinopathy or hypertensive chorioretinopathy could not be ruled out.

### Treatment

Twenty-four patients (31 eyes) with no central visual acuity loss at the initial visit who did not progress clinically were not treated and were followed thereafter. Fourteen patients (21 eyes) with progressive central visual acuity loss were treated as follows: intravenous methyl-prednisolone (PSL) at 1,000 mg/day for 3 days, oral PSL at 30 mg/day for 7 days, and intravenous methyl-PSL at 1,000 mg/day for 3 days. The PSL dose was then tapered as follows: 1 month at 30 mg/day, 1 month at 20 mg/day, 1 month at 15 mg/day, 1 month at 10 mg/day, and 1 month at 5 mg/day. In patients experiencing worsened visual field defects on perimetry or subjective symptoms at re-examination, the schedule was prolonged, or the dose was temporarily increased. Two patients experienced multiple recurrences when the oral PSL dose was decreased and were prescribed oral azathioprine at 50 mg/day in addition to corticosteroids.

### Ophthalmologic Examinations

During the initial visit, all patients underwent thorough ophthalmic examinations including a decimal BCVA assessment with a Japanese standard Landolt visual acuity chart, indirect ophthalmoscopy, FA, indocyanine green angiography (ICGA), OCT (OCT Ophthalmoscope C7, RS-3000, or RS-3000 Advance; Nidek, Gamagori, Japan), and 20J scotopic single-flash ERG, followed several days later by visual field testing (Goldmann perimetry and/or the Humphrey 30–2 Swedish interactive threshold algorism standard test) and multifocal ERG. Multifocal ERG was recorded with the Visual Evoked Response Imaging System (EDI, San Mateo, CA). The extent of anterior vitreous cells was quantified using the general grading score for aqueous inflammatory cells [19] and was classified as mild (occasional~1+ cells), moderate (2~3+ cells), and severe (4+ cells). When orbital or intracranial involvement was suspected at the initial visit, orbit and brain computed tomography or magnetic resonance imaging was performed. During OCT, a conventional cross-sectional B-scan through the fovea and/or the visual field defect site horizontally and vertically at a 6.0 mm × 6.0 mm scan length was performed. Serological testing and antibody titer evaluations for syphilis, toxoplasmosis, cytomegalovirus, herpes simplex virus, herpes zoster virus, serum antinuclear antibody, rheumatoid factor, anti-neutrophil cytoplasmic antibodies, angiotensin-converting enzyme, anti-thyroid peroxidase antibody, and anti-phospholipid antibody were performed as well. During follow-up, BCVA assessment, Humphrey perimetry, and OCT were performed to quantitatively evaluate the activity of AZOOR. Patients included in this study were classified into non-treated eyes and corticosteroid-treated eyes, and BCVA and mean deviation (MD) values on Humphrey perimetry were statistically analyzed between the worst initial (before treatment) and the final values in both groups.

### Statistics

The BCVA was converted to the logMAR scale for statistical analysis. The Wilcoxon signed-rank test was used to compare changes in the logMAR values of BCVA and MD values on Humphrey perimetry in eyes diagnosed with AZOOR. Simple linear regression analysis and a multiple stepwise linear regression analysis determined the independent variables affecting logMAR BCVA and MD values at the initial and final visits. *P* values less than 0.05 were considered statistically significant in all analyses. A significant improvement or deterioration in MD values during follow-up was defined as MD value changes of 30% or more.

## Results

### Patient Demographics

The Japanese patient population comprised 7 male (18.4%) and 31 female (81.6%) patients. Bilateral involvement was diagnosed in 7 patients (18.4%) at the initial visit and in 14 patients (36.8%) at the last visit. The mean presumed age of AZOOR onset was 33.2 ± 8.7 years (range, 15–47 years), and the mean age at the initial visit was 35.0 ± 10.6 years (range, 15–61 years). Presumed duration from the onset to the initial visit ranged from 0 to 122 months (mean, 14.1 ± 30.1; median, 1 month). The follow-up duration ranged from 6 to 132 months (mean, 37.7 ± 31.3; median, 31 months).

### Medical and Family History

Systemic diseases were noted in the medical history in 13 cases (34.2%). Pregnancy, Hashimoto disease, and hyperthyroidism were diagnosed in two cases each, and cervical cancer (after confirming no metastasis), thyroid cancer (after surgical excision, no metastasis), well-controled schizophrenia, rheumatoid arthritis, suspected Sjögren’s syndrome, atopic dermatitis, and chronic pancreatitis (idiopathic, no medication) were diagnosed in one case each. Two eyes in a single patient have been administered topical latanoprost for ocular hypertension. Seven cases (18.4%) had one or more family members with Type 1 diabetes mellitus, retinitis pigmentosa, systemic lupus erythematosus, Behçet's disease, Basedow’s disease, or polygrandular autoimmune syndrome.

### Subjective Symptoms

Centrally blurred vision and/or visual field defects at one or more zones were reported in 48 eyes (92.3%). Four eyes were only mildly affected by bilateral AZOOR and were asymptomatic. Thirty-one (59.6%) eyes had photopsia. Four (19.0%) of 21 patients questioned reported flu-like symptoms before the onset of AZOOR.

### Refraction

**Fig 1:**
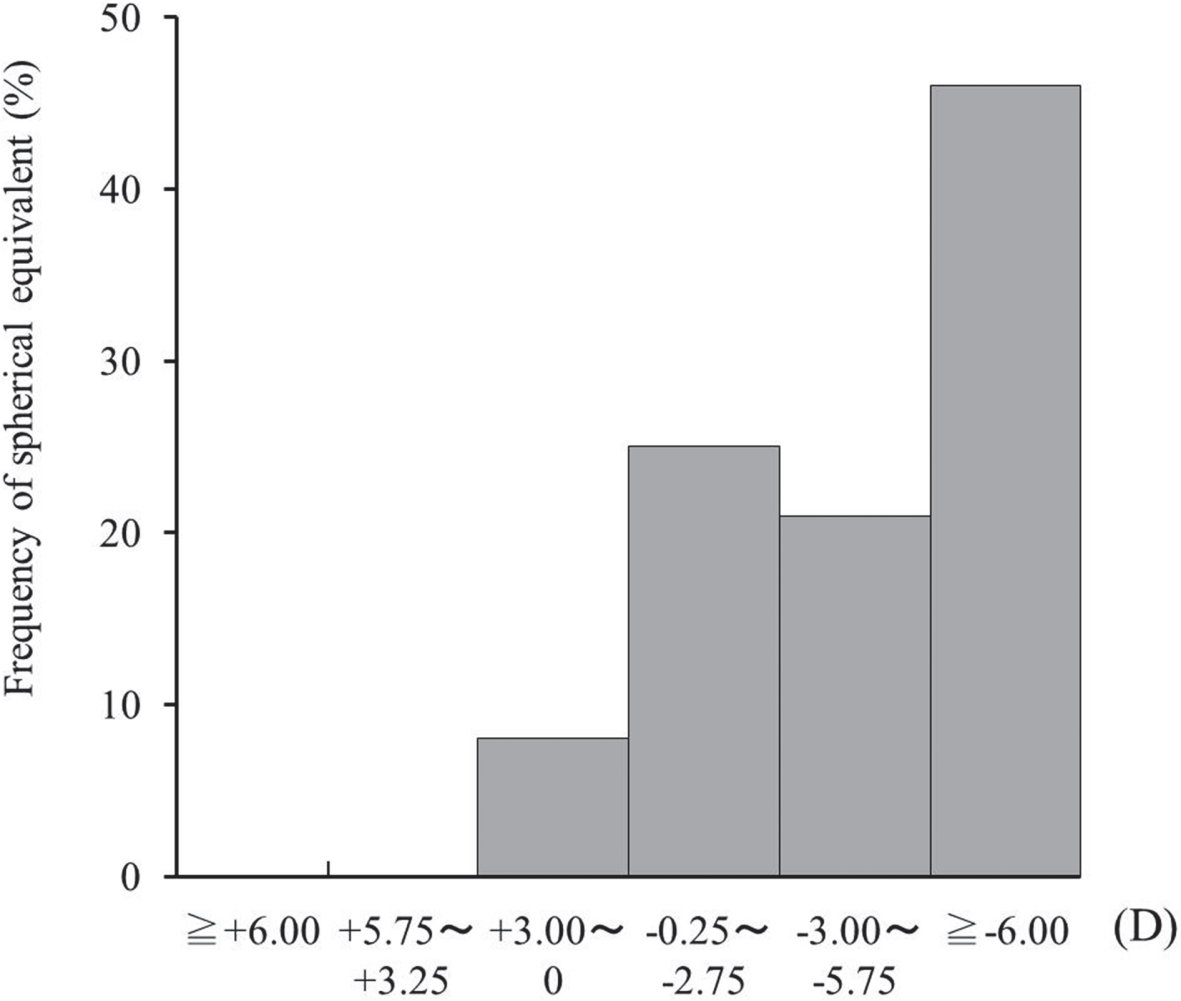
Frequency of spherical equivalent in 52 eyes with acute zonal occult outer retinopathy (AZOOR). Myopia of -6.0D or over was the most frequent (46.1%).

Of the 52 eyes, 48 (92.3%) were diagnosed with myopia: less than -3D in 13 eyes (25.0%), -3D to -5.75D in 11 eyes (21.2%), and greater than or equal to -6.0D in 24 eyes (46.1%) (Fig 1). Hyperopia or emmetropia was diagnosed in four eyes (7.7%).

### Ophthalmic Examinations

**Fig 2:**
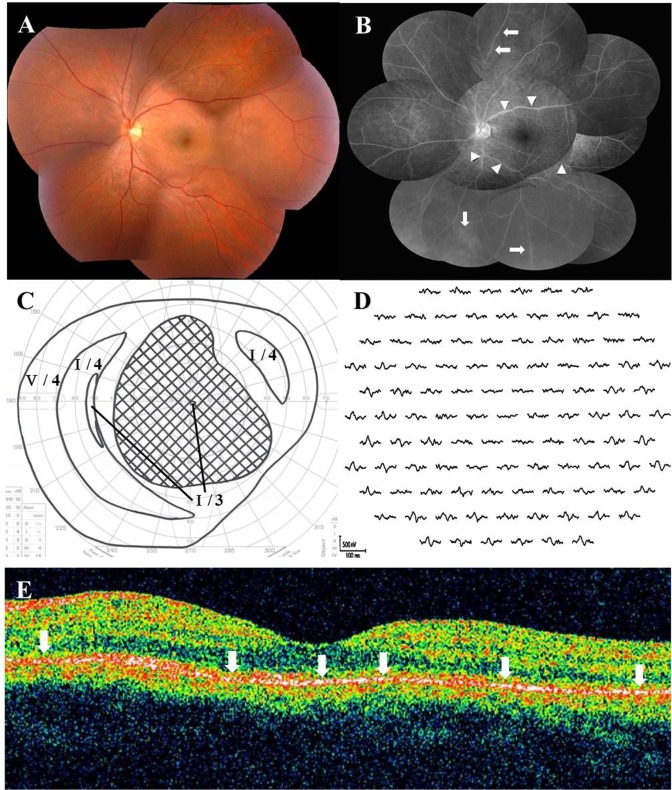
Photographs of the left eye at the initial visit in a 36-year-old male patient with AZOOR. (A) Fundus photograph shows normal appearance, except for retinal arterial narrowing. (B) Late-phase fluorescein angiography shows staining of the retinal vein walls (arrows) and leakages from the retinal vessels (arrowheads). (C) Goldmann perimetry reveals a central scotoma of 80 × 70°. LogMAR value of the best-corrected visual acuity (BCVA) decreased to 2.0. (D) Multifocal electroretinography (ERG) shows reduced responses corresponding to the visual field defect. (E) Horizontal optical coherence tomography through the fovea shows diffuse ellipsoid zone loss (arrows) in the macular area.

**Fig 3:**
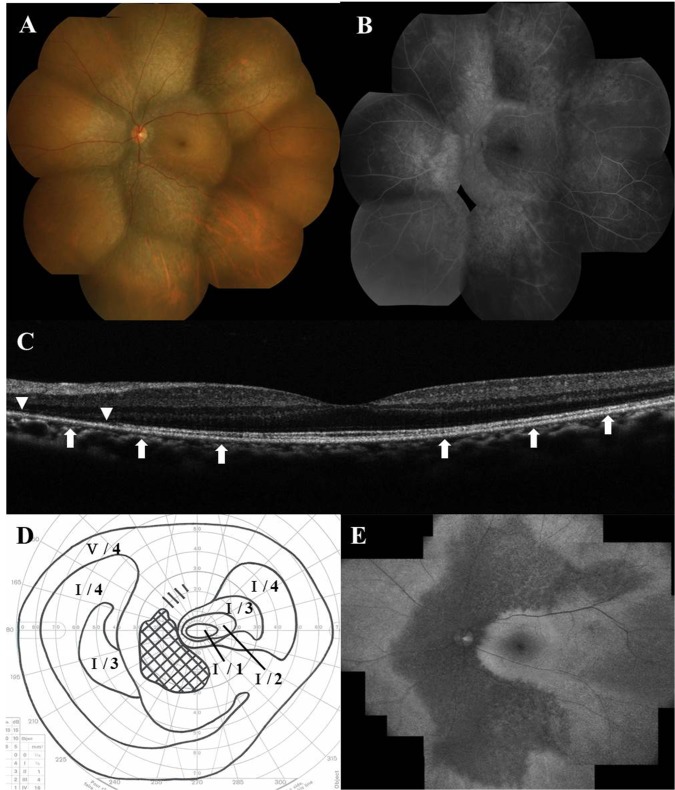
Photographs of the left eye 5 years (A–D) and 8 years (E) after systemic corticosteroid therapy in a patient shown in Fig 2. (A) Fundus photograph shows the development to zonal retinal atrophy at the retinal pigment epithelium level along the retinal arcade vessels. (B) Late-phase fluorescein angiography indicates the resolution of retinal vasculitis and the window defect corresponding to the zonal retinal atrophy. (C) Spectral-domain optical coherence tomography shows recovery of the ellipsoid zone and the interdigitation zone at the fovea, but the ellipsoid zone (arrows) and the interdigitation zone (arrowheads) are lost in the surrounding area. (D) Goldmann perimetry shows central scotoma shrinkage associated with central sensitivity recovery; the logMAR BCVA recovered to -0.18. (E) Fundus autofluorescence demonstrates decreased autofluorescence in the corresponding zonal retinal atrophy.

Relative afferent pupillary defect was positive in 12 (23.5%) of 51 eyes examined at the initial visit. Slit-lamp examination did not reveal any cells in the anterior chamber of any eye and mild (14 eyes) or moderate (8 eyes) cells in the anterior vitreous in 22 (44.9%) of the 49 eyes examined. Seven of 8 eyes with moderate anterior vitreous cells were administered systemic corticosteroid therapy. The retina was funduscopically normal in 43 eyes (82.7%) (Fig 2A), but myopia-associated punctuate or patchy chorioretinal atrophic lesions at the posterior pole in four eyes, a few punctate chorioretinal scars outside the posterior retinal pole in two eyes, and a lacquer crack lesion in two eyes were found. A white line at the margin of the affected area was observed in one eye with a large scotoma; the line spontaneously disappeared. Myopic temporal conus was found in 15 eyes, morning glory-like optic disc with no retinal schisis in one eye, and the sheathing, narrowing, and tortuosity of retinal arteries were observed in two eyes. During follow-up examinations, five eyes (9.6%) developed an area of zonal retinal degeneration at the RPE level with or without pigmentation, despite an initially normal retinal appearance (Fig 3A); however, none of the patients had an apparent demarcating line at the margin of the retinal degeneration.

### Fundus Angiography

**Fig 4:**
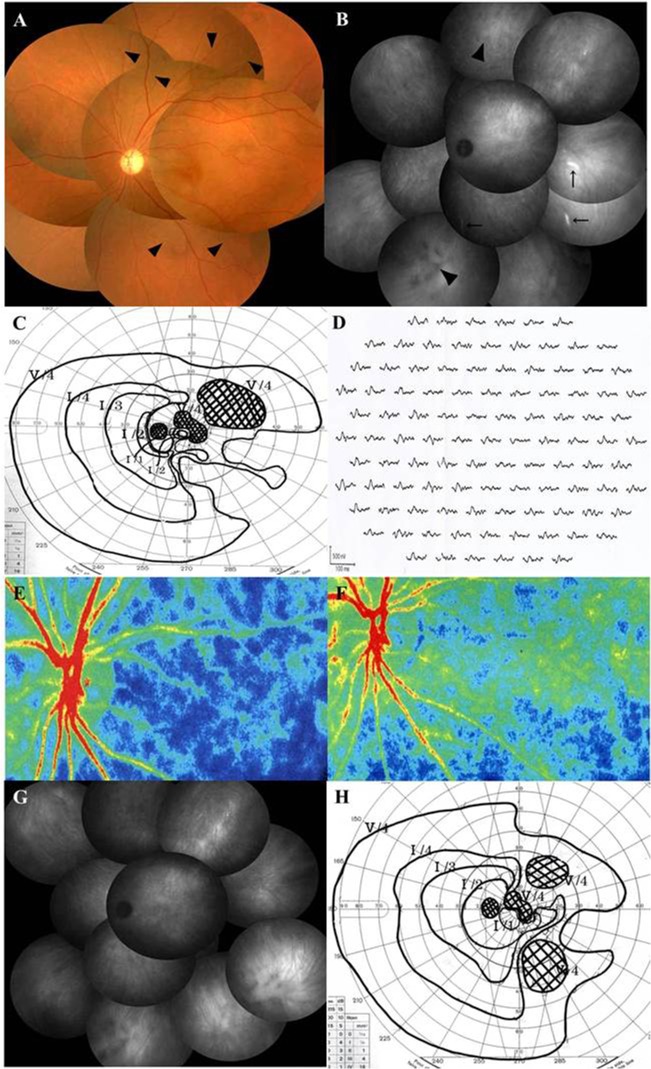
Photographs of the left eye before (A-E) and after (F-H) systemic corticosteroids therapy in a 39-year-old woman with AZOOR. (A) Fundus photograph shows normal retinal appearance except for narrowing and tortuosity of the retinal arteries (arrowheads). The patient’s logMAR BCVA was 0.52. (B) Indocyanine green angiography (ICGA) in the late phase shows hyperfluorescence along with choroidal middle or large vessels (arrows) and hypofluorescent patches (arrowheads). (C) Goldmann perimetry shows paracentral and isolated scotomata together with peripheral contraction. (D) Multifocal ERG reveals decreased responses corresponding to the visual field defects. (E, F) Composite color map of laser speckle flowgraphy. Mean blur rate (MBR), an index of relative blood flow velocity, increased three weeks after corticosteroid pulse therapy (F) compared to the pretreatment level (E), suggesting an improvement in choroidal circulation impairment after treatment. Warm color indicates high MBR and cool color low MBR. (G) On late-phase ICGA three weeks after treatment, hyperfluorecence along with choroidal vessels disappeared. Her logMAR BCVA improved to 0.10. (H) On Goldmann perimetry at 14 months after treatment, scotomata and peripheral contraction ameliorated with BCVA further improving to -0.18.

Initially, FA showed no abnormalities in 28 (56.0%) of 50 eyes examined. The optic disc staining and retinal vascular wall staining with leakage were observed on the late phase in 15 eyes (30.0%) and 13 eyes (26.0%), respectively (Fig 2B), and hyperfluorescent punctate lesions were identified in 4 eyes (8.0%). Of the 44 eyes examined with ICGA, 10 eyes (22.7%) appeared normal, but 29 eyes (65.9%) had a diffuse choroidal hyperfluorescence at the posterior pole to the mid-peripheral region during the middle phase. In addition, punctate-patchy hypofluorescence at the posterior pole to mid-peripheral region was observed in 17 eyes (38.6%) (Fig 4B), and the hyperfluorescence along with the choroidal middle or large vessels was seen in 6 eyes (13.6%) (Fig 4B).

### ERG Examination

In the 47 eyes examined, single-flash ERG showed a normal amplitude in 28 eyes (59.6%), reduced a-wave amplitude in 7 eyes (14.9%), reduced b-wave amplitude in 5 eyes (10.6%), reduced a- and b-wave amplitudes in 3 eyes (6.4%), and non-recordable responses in 4 eyes (8.5%). There were noticeably reduced multifocal ERG responses corresponding to visual field loss in all of the eyes examined (Figs 2D and 4D).

### OCT Examination

Forty-four of the 48 eyes that underwent OCT had ellipsoid zone irregularities corresponding to retinal sites with the visual field defect (Figs 2E and 3C). Among the eyes examined with SD-OCT, the interdigitation zone (originally called the cone outer segment tip line) was lost in four eyes [18], despite a visually normal ellipsoid zone.

### Systemic Screenings

Infectious disease tests showed normal results in all patients. Serum autoantibodies were detected in 8 patients (21.1%) as follows: anti-nucleus antibody (≧160×) in 5 patients, high levels of anti-thyroglobulin and/or thyroid peroxidase autoantibodies in 4 patients, rheumatoid factor in 3 patients, and anti-SS-A antibody in 1 patient (there is some overlap). Imaging of the orbit and brain revealed no abnormal findings in all 16 patients examined.

### Visual Acuity Changes

**Fig 5:**
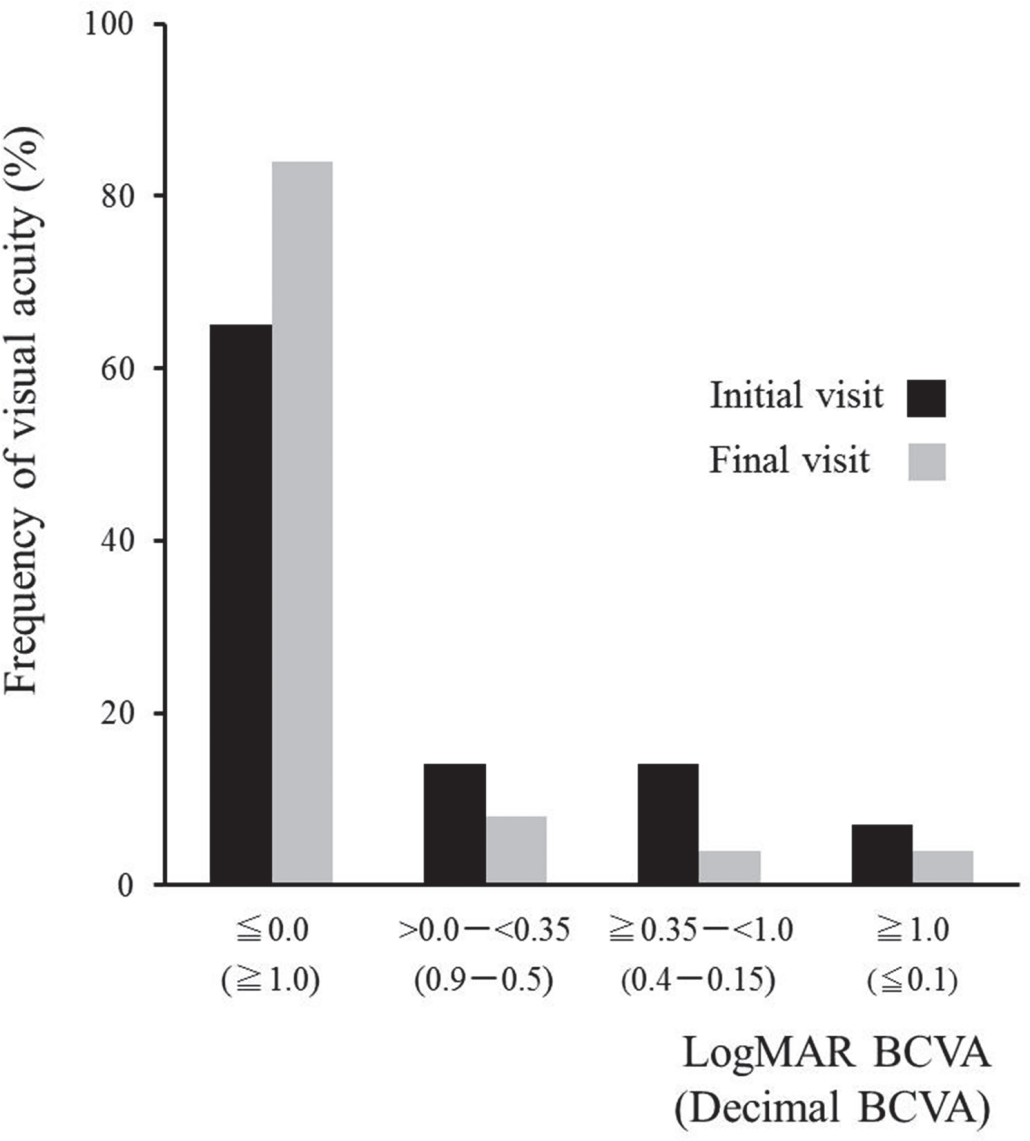
BCVA changes in 52 eyes with AZOOR. The final logMAR BCVA was 0.0 or less in 84.7% and 1.0 or more in only 3.8%.

The logMAR BCVA (*N* = 52) at the initial visit or before treatment was 0.0 or less in 34 eyes (65.3%), >0.0–<0.35 in 7 eyes (13.5%), ≧0.35–<1.0 in 7 eyes (13.5%), and 1.0 or more in 4 eyes (7.7%) (Fig 5). The BCVA at the final visit was 0.0 or less in 44 eyes (84.7%), >0.0–<0.35 in 4 eyes (7.7%), ≧0.35–<1.0 in 2 eyes (3.8%), and 1.0 or more in 2 eyes (3.8%). In all eyes (*N* = 52), the mean BCVA logMAR values at the initial and final visits were 0.10 ± 0.42 and -0.0035 ± 0.36, respectively. There was no significant difference between these phases (*P* = 0.06). In the 31 non-treated eyes, no statistical differences were found between the initial (-0.10 ± 0.11) and final logMAR BCVA (-0.11 ± 0.07) (*P* = 1.0). In contrast, in the 21 eyes administered systemic corticosteroids, the final BCVA (0.16 ± 0.52) was significantly better than the pre-treatment value (0.56 ± 0.60) (*P* = 0.007).

### Perimetry

Goldmann perimetry at AZOOR onset revealed an enlarged blind spot often associated with visual field defects of the other types in 26 eyes (50.0%), ring scotoma in 13 eyes (25.0%), arcuate scotoma in 4 eyes (7.6%), central scotoma (CS) in 3 eyes (5.8%), and other factors in 6 eyes (11.5%) (Figs 2C, 4C and Table 1).

**Table 1:** Visual Field Findings at Onset of AZOOR in 52 Eyes with AZOOR.

Types of Scotomata	Number of Eyes	%
BSE+isolated scotoma	10	19.2
BSE+central scotoma	7	13.5
BSE	6	11.5
BSE+contraction	3	5.8
Ring scotoma	13	25.0
Central scotoma	3	5.8
Arcuate scotoma	2	3.8
Arcuate scotoma+PCS	2	3.8
The others	6	11.5
Total	52	100

AZOOR = acute zonal occult outer retinopathy

BSE = blind spot enlargement; PCS = paracentral scotoma

**Fig 6:**
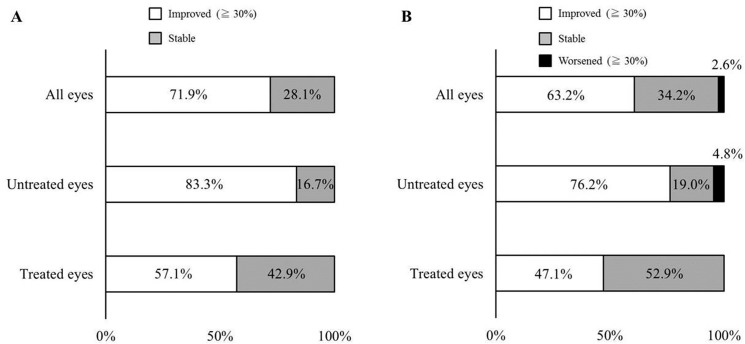
Changes in mean deviation (MD) values on Humphrey perimetry from baseline to 6 months (A) and the final visit (B). (A) MD values increased by 30% or more in 71.9% of all eyes (83.3% of untreated eyes and 57.1% of treated eyes). None of the eyes showed a worsening of 30% or more. (B) MD values increased by 30% or more in 63.2% of all eyes (76.2% of untreated eyes and 47.1% of treated eyes), but decreased by 30% or more in only 2.6% of all eyes.

Of 32 AZOOR eyes that received Humphrey perimetry at 6 month after baseline, the 6-month MD values increased by 30% or more in 23 eyes (71.9%) and remained unchanged in 9 eyes (28.1%), as compared to the baseline MD values (Fig 6A). None of the eyes showed a worsening of 30% or more in this short-term period. These 23 eyes achieving the early-phase improvement of visual field loss comprised 15 of 18 untreated eyes (83.3%) and 8 of 14 treated eyes (57.1%). The MD values at the final visit increased by 30% or more in 24 eyes (63.2%), remained unchanged in 13 eyes (34.2%), and decreased by 30% or more in 1 eye (2.6%) of all 38 eyes followed up using Humphrey perimetry (Fig 6B). In all eyes (*N* = 38), the mean MD at the final visit (-5.30 ± 8.51 dB) was significantly higher than the baseline (-9.31 ± 8.39 dB) (*P* = 0.000004). In the untreated eyes (*N* = 21), the mean MD at the final visit (-1.65 ± 2.85 dB) was significantly higher than the baseline (-5.80 ± 4.32 dB) (*P* = 0.00002). In eyes administered corticosteroids (*N* = 17), the mean MD at the final visit (-9.82 ± 10.74 dB) was significantly higher than the initial value (-13.66 ± 10.73 dB) (*P* = 0.02).

### Visual Function

As shown in Table 2, the initial visual function in AZOOR patients was positively correlated with the ERG a-wave amplitude at the initial visit, but negatively with the presence of moderate anterior vitreous cell infiltration at the initial visit. Visual function at the final visit was positively correlated with BCVA and MD at the initial visit and the ERG a-wave amplitude; it negatively correlated with moderate anterior vitreous cell infiltration, the presence of retinal atrophy at the final visit, and the administration of systemic corticosteroid therapy (Table 3). Multiple stepwise linear regression analysis revealed that moderate anterior vitreous cells, myopic severity, and a-wave amplitude significantly correlated with visual functions at the initial visit (Table 2). On the other hand, the initial MD, female sex, moderate anterior vitreous cells, and retinal atrophy significantly correlated with visual functions at the final visit (Table 3).

**Table 2:** Variables Affecting Visual Functions at Initial Visit in Patients with AZOOR.

	Initial BCVA (logMAR)				Initial MD			
	Simple linear regression analysis		Multiple linear regression analysis		Simple linear regression analysis		Multiple linear regression analysis	
	*cor*	*P value*	*β*	*P value*	*cor*	*P value*	*β*	*P value*
Age	0.20	0.1450	-	-	-0.028	0.8664	-	-
Sex	-0.0057	0.9681	-	-	-0.27	0.1055	-	-
Moderate ant.vit.cells	0.42	0.0024**	-	-	-0.61	0.0001**	-0.32	0.0216*
Degree of myopia	-0.19	0.1886	-	-	-0.14	0.3981	-0.24	0.0338*
Retinal vasculitis	0.029	0.8430	-0.20	0.1270	-0.042	0.8030	0.17	0.1445
a-wave in ERG	-0.60	6.9E-6**	-0.65	1.0E-5**	0.73	8.5E-7**	0.67	1.7E-5**

BCVA = best-corrected visual acuity; MD = mean deviation; ant.vit.cells = anterior vitreous cells; ERG = electroretinography

* *P* < 0.05;

** *P* < 0.01

**Table 3:** Variables Affecting Visual Functions at Final Visit in Patients with AZOOR.

	Final BCVA (logMAR)				Final MD			
	Simple linear regression analysis		Multiple linear regression analysis		Simple linear regression analysis		Multiple linear regression analysis	
	*cor*	*P value*	*β*	*P value*	*cor*	*P value*	*β*	*P value*
Initial VA (logMAR)	0.48	0.0004**	0.25	0.0939	-	-	-	-
Initial MD	-	-	-	-	0.81	1.0E-9**	0.91	1.3E-7**
Age	0.21	0.1401	-	-	-0.18	0.2915	-0.13	0.0921
Sex	-0.21	0.1410	-0.27	0.0399*	-0.16	0.3489	-	-
Moderate ant.vit.cells	-0.019	0.8955	-0.37	0.0207*	-0.36	0.0339*	0.54	2.3E-5**
Degree of myopia	0.081	0.5697	-	-	-0.15	0.3808	0.17	0.0549
Retinal atrophy	0.49	0.0002**	0.66	0.0002**	-0.74	1.4E-7**	-0.30	0.0340*
Retinal vasculitis	-0.036	0.8043	-0.27	0.0654	-0.11	0.5214	-0.15	0.0941
a-wave in ERG	-0.46	0.0012**	-	-	0.72	1.3E-6**	-	-
Treatment	0.37	0.0077**	-	-	-0.48	0.0024**	-0.15	0.1214

BCVA = best-corrected visual acuity; MD = mean deviation; ant.vit.cells = anterior vitreous cells; ERG = electroretinography

* *P* < 0.05;

** *P* < 0.01

### Recurrences and Ocular or Systemic Complications

AZOOR recurrence occurred in 9 eyes (17.3%) of 7 patients (18.4%) during follow-up. Six of these 7 patients had received systemic corticosteroid therapy. Four of the 6 treated patients experienced recurrences when oral PSL was tapered to less than 15 mg/ day; 2 cases were subsequently administered a second corticosteroid pulse therapy. The mean number of recurrences was 1.7 (range, 1–4), and the median duration from the onset to the recurrences was 6.5 months (3–52 months). Three patients were diagnosed with steroid-induced diabetic mellitus, and 1 patient experienced a pulmonary thrombosis and a lower extremity vein thrombosis. There were no ocular complications in the AZOOR eyes during follow-up. In 1 patient, idiopathic choroidal neovascularization occurred in the contralateral eye during follow-up.

## Discussion

In the present study, the clinical features and visual functional changes in a relatively large population of Japanese AZOOR patients with a follow-up duration of more than 6 months were retrospectively examined. Eyes with good visual acuity at the initial visit spontaneously showed good visual outcomes with significantly improved MD values. Eyes that developed progressive visual deterioration also showed significantly improved visual function after receiving systemic corticosteroid therapy. The final logMAR BCVA was <0.35 in 92.4% and ≧1.0 in 3.8% of patients. Retinal atrophy later developed in 9.6% of AZOOR eyes. A decreased MD at the initial visit, female sex, moderate anterior vitreous cells, and retinal atrophy were prognostic factors affecting visual function at the final visit. This is the first report that not only characterized the clinical features and visual function of Japanese AZOOR patients, but also elucidated the variables affecting visual functions in AZOOR.

Patients with good visual function at the initial visit accounted for 60% of the study population, and their perimetry results significantly improved without treatment, with visual acuity remaining stable during follow-up. These results suggest that visual function improves spontaneously in a certain percentage of Japanese AZOOR patients. Furthermore, in the remaining 40% of patients with progressive visual impairment, visual functions significantly improved after long-term high-dose systemic corticosteroid therapy. In a subset of these patients, chronic corticosteroid therapy was required because AZOOR recurred when the dose was decreased or the therapy was discontinued. It is difficult to determine the therapeutic efficacy of systemic corticosteroids in the enrolled patients because spontaneous visual improvement could not be ruled out in this retrospective study. However, recent reports found that AZOOR was successfully treated with immunosuppressive therapy including systemic corticosteroids [6,8,20] and adalimumab [21]. Randomized prospective studies of a large patient population are needed to clarify the efficacy of systemic corticosteroid therapy for AZOOR patients with progressive vision loss.

In this study, the ERG a-wave amplitudes at the initial visit were significantly correlated to the initial visual function. This result supports that AZOOR causes visual impairment by affecting the photoreceptor. Moreover, a moderate level of anterior vitreous cells negatively affected the initial visual function. Gass et al. reported that patients with anterior vitreous cells frequently have a high incidence of large visual field defect sites, subsequent retinal degeneration, and a lack of visual improvement [2]. Our results seem to verify these observations by Gass et al. and suggest that these patients had worse visual functions due to severe activity at the initial visit. Conversely, the presence of this finding positively affected the final visual functions. Interestingly, most of the eyes with moderate anterior vitreous cells received systemic corticosteroid therapy, leading to a significant improvement in visual functions. These seemingly contradictory results may possibly represent the efficacy of our systemic corticosteroid therapy regimen for AZOOR patients with severe activity.

Associated retinal atrophy also correlated with the severity of visual field impairment at the final visit. Gass et al. observed that visual acuity seemed to improve when the fundus maintained a normal appearance during follow-up [2]. Our result suggests that patients with this finding involve permanent visual field disorders following irreversible damage to the outer retina due to having severe activity.

Clinical features of the present study examining Japanese patients and the previous study of primarily Caucasian patients [2] were summarized in Table 4. There were no differences in the age at the onset and the gender ratio between both studies. The frequency of myopia was higher in Japanese patients; however, this may have reflected the higher incidence of myopia in Japan than in the United States. Notably, Japanese patients had a lower rate of retinal atrophy development, fewer patients with poor visual outcomes at final visit, and more patients with good visual outcomes at final visit, although the substantial gaps in the follow-up period (median: 31 months vs. 96 months) and the duration from the onset to the recurrence (median: 6.5 months vs. 39 months) between this study and the previous report [2] may have influenced the differences in these clinical parameters. Nevertheless, the early-phase improvement (over 70%) of visual field loss in our patients, whether treated or untreated, contrasted sharply with the tendency (roughly less than 30%) of short-term (within 6 months) amelioration of the visual field shown in the previous report [2]. Thus, Japanese AZOOR patients appear to present good visual functions at least in the relatively early course of the disease. Several mechanisms (i.e., ethnic difference, efficacy of systemic corticosteroids, and possibility of more frequent inclusion of milder cases in our study) would be speculated as the reason(s) of the differences observed. Further studies are needed to make a proper comparison of the clinical features and visual prognosis between Japanese and Caucasian AZOOR patients by matching the follow-up duration and treatment regimens.

**Table 4:** Clinical Features of the Present Study and a Previous Study.^#^

Variables	Saito et al.	Gass et al.
Patients Number	38 Patients (52 Eyes)	51 Patients (90 Eyes)
Race	Japanese	Caucasian
Follow-up duration (months)*	31 (6–132)	96 (36–420)
Age at onset (years) *	35 (15–47)	33 (13–63)
Female patients	31/38 (82%)	37/51 (73%)
Unilateral involvement at initial visit	31/38 (82%)	31/51 (61%)
Unilateral involvement at final visit	24/38 (63%)	12/51 (24%)
Myopia	48/52 (92%)	59/90 (66%)
Photopsia	31/52 (60%)	69/90 (77%)
Recurrences	7/38 (18%)	16/51 (31%)
Normal or AZOOR-unrelated fundus at initial visit	49/52 (94%)	82/90 (91%)
Retinal atrophy at final visit	5/52 (10%)	43/90 (48%)
Final logMAR BCVA <0.35	48/52 (92%)	61/90 (68%)
Final logMAR BCVA ≧1.0	2/52 (4%)	24/90 (27%)

^#^ Gass JDM et al., Am J Ophthalmol 2002;

* Median (range);

BCVA = best-corrected visual acuity

## Conclusions

In the present study, approximately 60% of enrolled Japanese patients with AZOOR had good visual acuity at the initial visit and these most showed spontaneous regression. In patients experiencing progressive visual impairment, visual function significantly improved after systemic corticosteroid therapy. The final logMAR BCVA was 0.0 or less in 85% of patients. Initial MD value, gender, moderate anterior vitreous cells, and retinal atrophy were prognostic indicators of the final visual function. Thus, Japanese patients with AZOOR appear to have good visual outcomes during the follow-up period of this study.
